# Anti-inflammatory effect of dental pulp stem cells

**DOI:** 10.3389/fimmu.2023.1284868

**Published:** 2023-11-23

**Authors:** FenYao Li, XinXin Wang, Jin Shi, ShuTing Wu, WenBo Xing, Yan He

**Affiliations:** Institute of Regenerative and Translational Medicine, Tianyou Hospital, Wuhan University of Science and Technology, Wuhan, China

**Keywords:** dental pulp stem cells, inflammation, anti-inflammatory, immune regulation, regeneration

## Abstract

Dental pulp stem cells (DPSCs) have received a lot of attention as a regenerative medicine tool with strong immunomodulatory capabilities. The excessive inflammatory response involves a variety of immune cells, cytokines, and has a considerable impact on tissue regeneration. The use of DPSCs for controlling inflammation for the purpose of treating inflammation-related diseases and autoimmune disorders such as supraspinal nerve inflammation, inflammation of the pulmonary airways, systemic lupus erythematosus, and diabetes mellitus is likely to be safer and more regenerative than traditional medicines. The mechanism of the anti-inflammatory and immunomodulatory effects of DPSCs is relatively complex, and it may be that they themselves or some of the substances they secrete regulate a variety of immune cells through inflammatory immune-related signaling pathways. Most of the current studies are still at the laboratory cellular level and animal model level, and it is believed that through the efforts of more researchers, DPSCs/SHED are expected to be transformed into excellent drugs for the clinical treatment of related diseases.

## 1 Introduction

**Figure 1:**
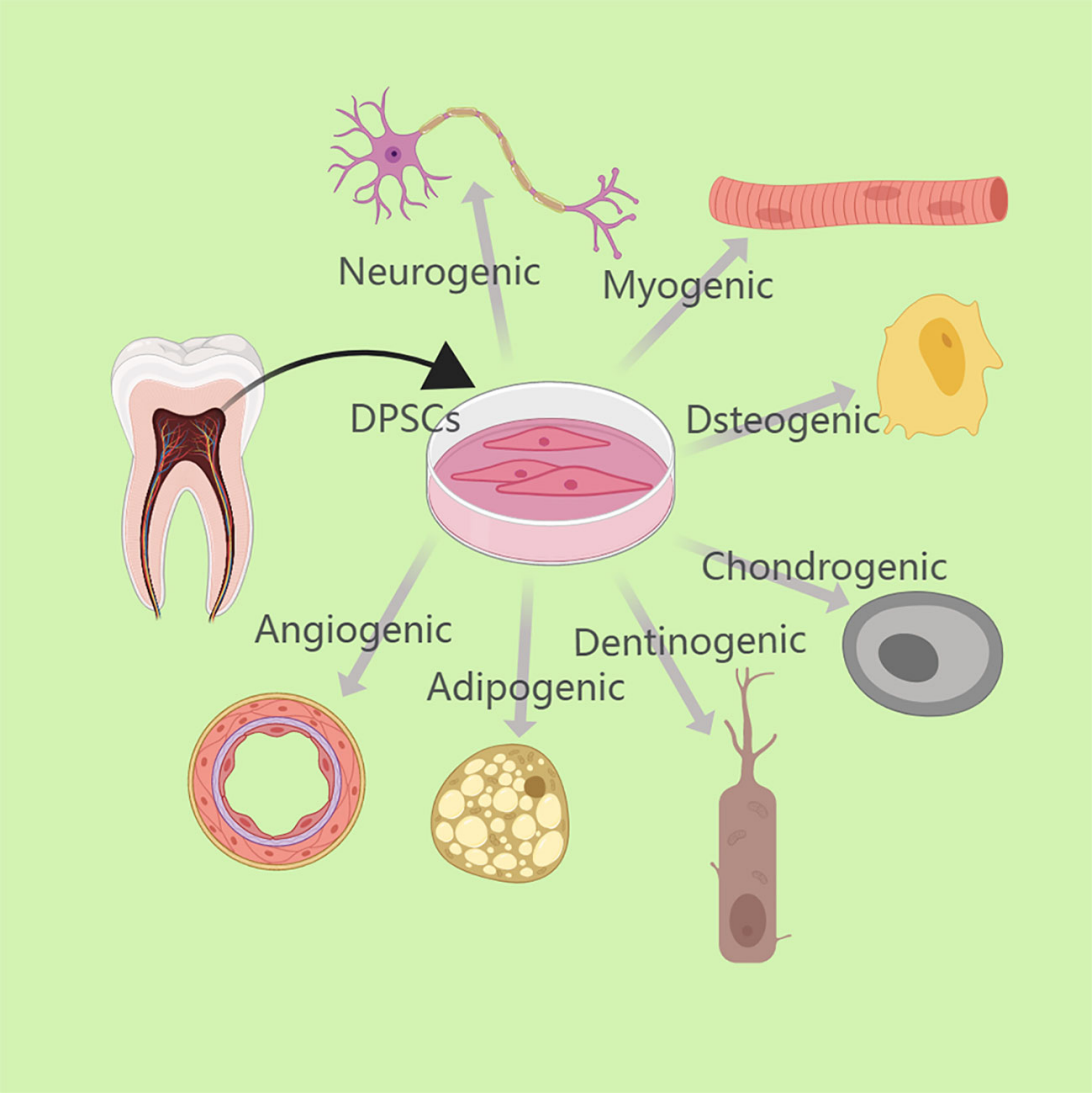
DPSCs can typically be obtained from pulp tissue of blocked wisdom teeth or orthodontic decimated teeth. Under particular circumstances, DPSCs can differentiate *in vitro* into dentinogenic cells, osteoblasts, chondrocytes, adipocytes, endothelial cells, neurons, etc.

Adult dental pulp stem cells (DPSCs) and stem cells from human exfoliated deciduous teeth (SHED) are self-renewing mesenchymal stem cells (MSCs) present in the perivascular area of the dental pulp (1, 2). DPSCs can typically be obtained from pulp tissue of blocked wisdom teeth, orthodontic decimated teeth (3). SHED can be extracted from pulp tissue of deciduous milk teeth of children aged 6-12 years (2). These cells are generally believed to originate from the cranial neural crest and can express early markers of bone marrow mesenchymal stem cells and neural ectodermal stem cells (4). These cells have grown in significance in the field of regenerative medicine due to their capacity for self-renewal, flexibility, high proliferation, and other potentials (5). Similar to bone marrow mesenchymal stem cells, they are able to differentiate into dentin-forming cells, osteoblasts, chondrocytes, adipocytes, endothelial cells, and neurons *in vitro* under specific conditions (6). (**Figure 1**) More importantly, they can modulate a variety of immune cells such as T lymphocytes and B lymphocytes, dendritic cells (DCs) and natural killer (NK) cells, thus becoming effective immunomodulators that inhibit pro-inflammatory processes and stimulate anti-inflammatory mechanisms (7, 8).

Inflammation is the first response that occurs after an injury or infection and is an immune defense process of the body (9). If the inflammatory process continues for too long, an excessive number of activated cells are drawn to the site of the damage and release an excessive amount of enzymes, chemokines, etc., which is harmful to tissue repair and promotes further deterioration (7). Early inflammatory response management may be essential for regeneration.

**Figure 2:**
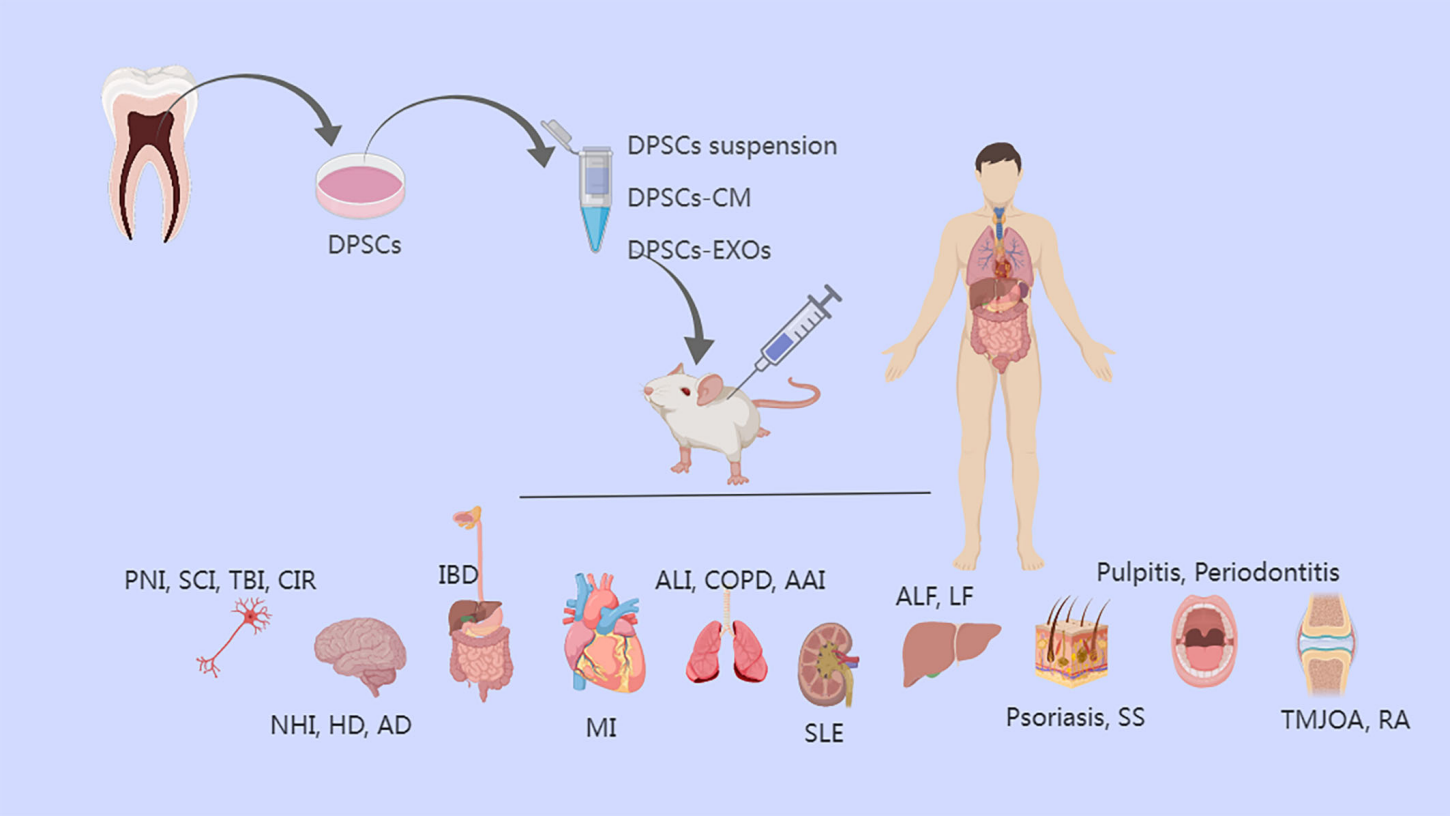
The treatment of diseases of various tissues/organs by anti-inflammatory effects of DPSCs and DPSCs-EXOs and DPSCs-CM. DPSCs, dental pulp stem cells; CM, conditioned medium; EXOs, exosomes; PNI, peripheral nerve injury; SCI, Spinal cord injury; TBI, traumatic brain injury; CIR, cerebral ischemia-reperfusion; NHI, neonatal hypoxia-ischemia; HD, Huntington’s disease; AD, Alzheimer’s disease, IBD, inflammatory bowel disease; MI, myocardial infarction; ALI, acute lung injury; COPD, Chronic Obstructive Pulmonary Disease; AAI, allergic airway inflammation; SLE, systemic lupus erythematosus; ALF, acute liver failure; LF, liver fibrosis; SS, Sjogren’s syndrome; TMJOA, temporomandibular joint osteoarthritis; RA, rheumatoid arthritis.

The innate immune system is associated with the resolution of inflammation, and the adaptive immune system also contributes significantly to this process. The expression of several proteins and cytokines, the recruitment of numerous immune cells at various times and in varying numbers, and various signaling pathways all play major roles in the regulation of the regenerative process (10, 11). Excessive inflammation can develop in a variety of conditions, including supraspinal nerve inflammation, neonatal hypoxia-ischemia, pulmonary airway inflammation, systemic lupus erythematosus, and diabetes (8, 12). Controlling and eliminating inflammation and modulating immunity will help regeneration and contribute to the treatment of these diseases (**Figure 2**).

Clinical practice today favors oral or local injections of medications like nonsteroidal anti-inflammatory medicines, corticosteroids, etc. to treat disorders associated to inflammation (13). To achieve anti-inflammatory effects in a short period of time and to be applied to most types of inflammation, glucocorticoids are used in clinical practice (14). Glucocorticoids have a rapid, powerful, non-specific anti-inflammatory effect and are effective in a wide range of inflammatory conditions. However, all kinds of drugs have their limitations, and the side effects of drugs may make the benefits outweigh the disadvantages (9, 15). For example, long-term use of glucocorticoids can reduce the body’s resistance to pathogenic microorganisms, induce or aggravate infections, induce ulcers, aggravate mental illness, and cause osteoporosis, muscle atrophy, and delayed wound healing (15, 16).

In comparison to traditional MSCs, DPSCs are more proliferative, anti-inflammatory, and anti-fibrotic (17, 18). Proteomics was employed by Bousnaki et al. to examine the anti-inflammatory potential of the secretome of DPSCs under various settings, and they came to the conclusion that DPSCs are a promising therapeutic tool for the treatment of inflammatory illnesses (19). DPSCs may be as effective as glucocorticoids for a variety of inflammatory conditions, but are safer and more favorable to regeneration (20, 21).

Currently, more studies are focusing on the anti-inflammatory and immunomodulatory roles of DPSCs (22). In this review, we will discuss the current research progress on the anti-inflammatory effects of DPSCs in various organ- and tissue-related diseases, as well as the understanding of the related anti-inflammatory mechanisms and immunomodulatory functions, which will help the future clinical application of DPSCs.

## 2 Effects of DPSCs on nerve-associated inflammatory diseases

Peripheral nerve injury (PNI) can cause acute or chronic neuropathic pain. Neuropathic pain, characterized by sensory and emotional disturbances, is also known as a neuroimmune disorder and is the result of the interaction between neurons and inflammatory mediators of immune cell/immune cell origin, particularly chemokines and cytokines, as well as histamine, adenosine triphosphate and prostaglandins (23, 24). PNI induces the release of certain inflammatory mediators at the site of injury, in the dorsal root ganglion, and in spinal cord receptor segments, and also induces the release of inflammatory mediators in the brain, causing supraspinal nerve inflammation (25). Chronic inflammation also activates microglia, with elevated reactive oxygen species and Nuclear Factor-κ-gene Binding (NF-κB) activity and dysregulated homeostasis, accompanied by the release of pro-inflammatory cytokines, leading to neurodegeneration and altered synaptic transmission (26). In a study, researchers evaluated the alleviating effects of DPSCs and neuronal differentiated DPSCs on PNI-induced neuro-inflammation in a rat model of sciatic nerve damage (12). Microglia that had been activated by sciatic nerve injury produced more reactive oxygen species, which increased downstream NF-κB activation and started the transcription of pro-inflammatory cytokines by nuclear translocation. The expression of pAMPK and SIRT1 was decreased, and the pro-inflammatory responses were amplified while anti-inflammatory components were downregulated. By migrating to the site of injury and starting immune and anti-inflammatory effects, decreasing microglia activation, down-regulate pro-inflammatory markers, and up-regulate pAMPK/SIRT1, DPSCs may effectively balance the inflammation of the supraspinal nerve caused by PNI as well as alleviate pain and promote rapid regeneration (27–29).

Besides PNI, the central nervous system injury is also worthy of attention. Spinal cord injury (SCI) is a traumatic disease of the central nervous system in which a series of cellular responses, such as inflammation and apoptosis, occur near the site of injury due to increased release of biomolecules such as interleukin (IL)-6 and tumor necrosis factor (TNF)-α, as well as some other cytokines (30–32). Albashari et al. focused their attention on DPSCs when investigating treatments for SCI. Their work, in which Pluronic F-127, a biosynthetic hydrogel, was mixed with DPSCs and basic fibroblast growth factor and applied at the site of SCI, showed that it not only promoted tissue regeneration at the site of injury, but also contributed to the recovery of sensory and motor functions, which is inextricably linked to the low inflammatory microenvironment provided by DPSCs (33). DPSCs express IL-6, IL-8, and TGFβ via the Toll-like receptor (TLR) during the neural inflammation phase in treating central nerve diseases (34). Expression of IL-8 is increased in DPSCs in spinal cord injury, and IL-8 is associated with maintenance of neuronal cell integrity and reduction of injury (35). DPSCs modulate the immune response after nerve injury by increasing the release rate of anti-inflammatory factors IL-10 and transforming growth factor (TGF)-β and decreasing the release rate of pro-inflammatory factors IL-6 and TNF-α (36). The NF-κB pathway plays a key role in pro-inflammatory factor-induced neuronal cell inflammation and a central role in pro-inflammatory factor-induced apoptosis (37). At acute SCI stage, pro-inflammatory factors and NF-κB signaling pathways are activated, causing severe neuro-inflammation, and microglia are active and act as immune cells in direct contact with nerve cells (38, 39). DPSCs attenuate the inflammation of SCI by decreasing NF-κB expression. It is possible to treat SCI with tropical use of DPSCs by reducing inflammation.

Similar to SCI, traumatic brain injury is a trauma-induced disease of the central nervous system. It is caused by an external mechanical force striking the skull and causing intracranial damage to neurons, axons, glial cells, and blood vessels. Next, the blood-brain barrier is disrupted, which causes neuro-inflammation, oxidative stress, and excitotoxicity, which causes additional neuronal damage, primarily at the site of the injury and nearby tissues (40). And in the long term this secondary damage may even cause lesions in areas of the brain far from the site of injury or may be referred to as a neuro-degeneration (41). When traumatic brain injury occurs, to protect the site of injury from pathogens, an endogenous inflammatory response is activated and proliferating immune cells secrete various chemokines, pro-inflammatory cytokines such as TNF-α, IL-1β, IL-6, and IL-12, and other inflammatory mediators (42). Due to the immunomodulatory properties of MSCs, which can effectively suppress neuro-inflammation, it has been shown that stereotactic or intravenous administration of MSCs can reduce the levels of various pro-inflammatory cytokines, such as IL-1β, IL-6, IL-17, interferon (IFN)-γ or TNF-α, in the serum and brain after brain injury (43, 44). Its powerful anti-inflammatory effect inhibits the NF-κB signaling pathway, which facilitates the treatment of secondary brain injury (43). Importantly, investigators found that MSCs significantly altered blood-brain barrier function by decreasing endothelial permeability, increasing VE-cadherin expression and VE-cadherin/β-catenin interaction, which may be a doorway to break through multiple neurological diseases (45). DPSCs are derived from neural crest cells, which can induce apoptosis of activated T cells *in vitro* and modulate immune responses, and the application of DPSCs to resist neuro-inflammation is promising for the treatment of traumatic brain injury (43).

In addition to traumatic brain injury and SCI, many diseases of the central nervous system: Huntington’s disease (HD), Alzheimer’s disease, Parkinson’s disease, neuro-inflammatory multiple sclerosis, etc., are characterized by varying degrees of inappropriate inflammatory/immune responses and tissue destruction (8, 46). Among them, HD is a progressive neurodegenerative disease that can be fatal with autosomal dominant inheritance. The current consensus is that striatal degeneration due to chromosomal abnormalities leads to lateral ventricular effusion, and the main case is characterized by neuronal loss and neuro-inflammation within the striatum and cortex, resulting in a range of motor and cognitive symptoms (47). In the clinic, HD can be diagnosed, but there is no very clear treatment for this disease. Some scholars have then focused on the possibility of MSCs treatment for it. In a prior study, Eskandari et al. used transplanted DPSCs to treat a 3-nitropropionic acid rat model of HD. These cells were placed in the mid-posterior region of the rat striatum and were able to survive and treat motor dysfunction (48). DPSCs treatment inhibited gliosis and microgliosis in the striatum and reduced the expression of inflammatory cytokines at the mRNA level. This action of DPSCs is caused in part by their capacity to release neurotrophic substances, to reduce apoptosis, and to suppress the expression of inflammatory cytokines (4, 49). Treatment based on DPSCs seems to provide effective treatment for HD and other neurodegenerative diseases, and although this treatment has not yet been translated clinically, it is well worth looking forward to (50).

There is growing evidence that inflammatory processes are involved in the pathogenesis of diabetic neuropathy, which can occur in various parts of the peripheral and central nervous system (51, 52). Diabetes is considered to be a chronic low-grade inflammatory disease, manifested primarily by the transduction of various inflammatory signals and the release of molecular products that generate and maintain neurological inflammation through the activation of mononuclear phagocytes, including resident microglia and blood-derived macrophages (53, 54). Omi et al. discovered that injection of skeletal muscle DPSCs significantly decreased the number of sciatic nerve macrophages in diabetic rats while increasing the gene expression of M2 macrophages and decreasing that of M1 macrophages. This resulted in the treatment of diabetes-induced sciatic nerve inflammation, an improvement in the conduction velocity and blood flow of the sciatic nerve, and an increase in intraepidermal nerve fiber density (55). In conclusion, the anti-inflammatory effect of DPSCs by modulating the ratio of M1/M2 macrophages may be an effective treatment for diabetic polyneuropathy.

Based on the anti-inflammatory effects of DPSCs in neuro-inflammation above through multiple pathways, these chronic diseases causing neuro-inflammation may consider the application of DPSCs, not necessarily as the only treatment, but as an adjuvant or collaborative way to combat such diseases by counteracting inflammation, achieving relief of disease symptoms and relieving chronic pain.

Neonatal hypoxia-ischemia (NHI), also known as neonatal hypoxic-ischemic encephalopathy, can lead directly to neonatal death (56). Even if they live, some neonates may have one or more neuropsychiatric conditions, such as cerebral palsy, mental retardation, or learning difficulties (57). When the fetal blood-brain barrier is not fully developed, if intrauterine infection occurs, inflammatory cells and toxins will cross the barrier and enter the brain tissue, which will not only cause inflammation in the endothelial tissue of the brain, but also stimulate the production of inflammatory cytokines such as IL-1β, IL-6 and TNF-α, thus aggravating the condition of hypoxic-ischemic encephalopathy (58). Existing treatment modalities can partially improve the neurological deficits of NHI, but there is no effective clinical treatment for this devastating disease. Chiu et al. used human DPSCs to treat a hypoxia-ischemia model in rats and found that DPSCs in NHI decreased the expression of pro-inflammatory factors (IL-1β, IL-6, TNF-α, INF-γ) and increased the expression of anti-inflammatory factors (IL-10, TGF-β) (57). This suggests that the anti-inflammatory effects of DPSCs synergize with effects such as neuro-regeneration to promote neuroplasticity and improve neurological outcome for a desired improved prognosis (59, 60). DPSCs may provide a viable strategy for restoring NHI-induced disability.

## 3 Effects of DPSCs on intestinal-associated inflammatory diseases

Inflammatory bowel disease (IBD) is an umbrella term for Crohn’s disease and ulcerative colitis, and is closely related to genetics, flora, and immunity (61, 62). Pathogenic and benign commensal bacteria coexist in the gastrointestinal environment, and the human immune system regulates intestinal inflammation and tolerance. When this balance is upset, chronic inflammation and the development of unfavorable innate and adaptive immune mechanisms occur, which then cause IBD (63). Barrier disruption leads to altered intestinal flora, which leads to abnormal activation of the mucosal immune system and is an important factor in the development of IBD (64). Furthermore, misregulated Th cell responses play a central role in the progression of chronic inflammatory processes in the gut (65). It has been shown that in the intestinal mucosa of healthy mice, CX3CR1+ macrophages inhibit IL-17 production, express anti-inflammatory molecules such as IL-10, and induce differentiation of Foxp3+ regulatory T (Treg) cells (66). CD103+ DCs effectively induce Treg cells to suppress inflammation by producing IL-10, TGF-β and retinoic acid in intestinal inflammation (67). Elevated number of Th17 cells and upregulation of IL-17 in mucosa of IBD patients compared to normal mucosa (68).

Multiple researchers have used systemic infusion of DPSCs to treat DSS-induced colitis in mice. DPSCs significantly decreased inflammatory cell infiltration and downregulated inflammatory cytokines in the colon, ameliorating colonic transmural inflammation, which in turn resulted in a reduction in wall thickness, an inhibition of epithelial ulceration, and the restoration of normal intestinal architecture (69). In their study, they also found that DPSCs’ ability to inhibit T-cell viability *in vitro* was decreased when the transmembrane protein Fas ligand (FasL) was knocked down. This could have prevented DPSCs from inducing T-cell apoptosis, which would have reduced their ability to improve the colitis phenotype. This finding suggests that FasL may be necessary for DPSC-mediated immune regulation (69, 70). The study by Földes et al. also focused on a DSS-induced mouse model of colitis and used intravenous injections of human DPSCs to study the effects on IBD (8). Their results showed that a single intravenous injection of human DPSCs can beneficially modify the development of acute colitis *in vivo*, confirming the protective role of human DPSCs in experimental colitis. Different species sources of DPSCs and different culture methods may lead to different biological activities; exogenous and endogenous cytokine levels and the timing of administration may also affect the efficacy of DPSCs (71). For example, pre-stimulation with INF-γ and TNF-α significantly enhanced the protective effect of DPSCs against colitis in mice (72, 73). In conclusion, DPSCs’ contribution to intestinal inflammation should not be understated based on their immunomodulatory and anti-inflammatory actions. Although the mechanism needs to be clarified further, the outlook is generally favorable.

## 4 Effects of DPSCs on lung-associated inflammatory diseases

Acute lung injury (ALI) progresses to a severe stage called acute respiratory distress syndrome (ARDS), characterized by a variety of changes caused by lung injury, including increased pulmonary capillary permeability, enhanced inflammatory cell infiltration, and diffuse alveolar and interstitial edema (74). When paraquat enters the human body, it causes ALI, which develops into ARDS. As the disease progresses, pulmonary edema and hemorrhage occur along with the infiltration of inflammatory cells into the interstitium and alveoli of the lungs, fibrosis, and eventually respiratory failure, which results in death (75, 76).

Regulating and reducing inflammation is an extremely important therapeutic strategy for ALI and ARDs due to paraquat toxicity. Geng et al. focused on this problem with the idea of using stem cells to act on this ALI-induced inflammation and subsequent lung fibrosis, and their work concluded that DPSCs have more potential as a treatment for ALI by comparing the anti-inflammatory effects of umbilical cord mesenchymal stem cells and DPSCs (77). Using hepatocyte growth factor (HGF), a multifunctional cytokine that affects multiple pathophysiological processes involved in inflammatory and immune responses, modified DPSCs more significantly inhibit inflammatory mediators, maintain alveolar structural integrity, and attenuate interstitial hemorrhage and inflammatory cell infiltration in the lung (77, 78). Supporting the repair of ALI by this means of relieving inflammation also can achieve the goal of reducing mortality due to paraquat toxicity.

Additionally, exposure to some allergens, such as dust, can cause allergic airway inflammation, and eosinophil infiltration into the lungs can result in lung inflammation. The work of Laing et al. used egg yolk OVA to induce allergic lung inflammation in a mouse model. OVA sensitization and subsequent aerosol stimulation resulted in intense allergic airway inflammation with eosinophil infiltration into the lungs. By injecting live/apoptotic DPSCs, the number of inflammatory cells in the lung lavage fluid of mice decreased, demonstrating that apoptotic DPSCs are effective in controlling lung inflammation (79). Indeed, apoptotic cells and apoptotic cell fragments are known to contribute to the adoption of an immunosuppressive phenotype by antigen-presenting cells (80, 81). Injection of pre-killed DPSCs may be a more effective, clinically simpler and safer alternative.

Chronic Obstructive Pulmonary Disease (COPD) encompasses a wide range of diseases with common functional characteristics, such as chronic bronchitis, chronic respiratory failure and emphysema (82). Gao et al. used intratracheal injection to transplant DPSCs into a mouse model of emphysema for a therapeutic investigation on chronic obstructive pulmonary disease. They discovered that DPSCs improved lung function and emphysema-like changes in COPD by activating the Nrf2 signaling pathway and decreased IL-1β, TNF-α, and IL-6 levels in lung tissue and bronchoalveolar lavage fluid (83).

People will inevitably suffer from inflammation of the lungs and airways due to environmental pollution, work factors, personal habits and other factors. The long lasting chronic inflammatory response leads to destruction of the alveolar wall and thinning of the alveolar sac, which in turn causes breathing difficulties and reduced lung function, such as COPD, bronchopulmonary dysplasia, ARDS, asthma, pulmonary fibrosis and pulmonary hypertension (84, 85). Exploring a safe and effective therapeutic strategy to reduce inflammatory damage in the lung is a major topic to be addressed in the field of lung disease research. MSCs suppress deleterious immune responses and differentiate into alveolar epithelial type II cells *in vitro* to suppress inflammation (86). As a type of MSCs, DPSCs modulating lung inflammation and related immune functions are being noticed by more and more researchers, and there is reason to believe that there will be more and more evidence of the importance of the anti-inflammatory effects of DPSCs.

## 5 Effects of DPSCs on kidney-associated inflammatory diseases

As a typically fatal autoimmune disease, nearly half of patients with systemic lupus erythematosus develop complications of lupus nephritis (87). Kidney injury occurs, immune complexes are deposited in glomeruli, tubules, and microvessels, and clinically occult nephritis, nephritis syndrome, and nephrotic syndrome occur. The existing treatment methods mainly include immunosuppression, glucocorticoids, dialysis treatment and kidney transplantation. Some patients with lupus nephritis have no effect on both glucocorticoids and immunosuppressants, and long-term use of immunosuppressants has obvious toxic side effects, such as ovarian failure, secondary infection and secondary malignancy (88, 89). The immunomodulatory effect of MSCs can intervene and block the pathogenesis of this autoimmune disease, and the therapeutic effect and prospect are more advantageous than other diseases (90). In their study, Tang et al. treated mice with lupus nephritis using DPSCs; the mice showed a reduction in IgG and IgM deposition in the glomeruli, a significant decrease in the number of CD4+ T cells that produce IFN-γ, and a reduction in perivascular inflammatory infiltrates (91). It has been shown that MSCs transplantation in lupus nephritis alters the ratio of T cell subsets, upregulates the proportion of Treg cells, downregulates IL-17 and TGF-β, and increases Foxp3, thereby suppressing renal inflammation and improving disease conditions (92). MSCs also exhibit inhibition of secretion of multiple pro-inflammatory factors by B cells, as well as suppression of dendritic cell development through secretion of IL-6 and prostaglandin E2 maturation, inhibit the proliferation and activation of CD8+ T cells, and stimulate CD4+ T cells to produce Th2 responses, upregulate Treg cells, and attenuate the inflammatory response associated with lupus nephritis (93). It is believed that DPSCs can play a role in the treatment of systemic lupus and lupus nephritis.

## 6 Effects of DPSCs on skin-associated inflammatory diseases

Psoriasis is a chronic relapsing inflammatory skin disease that can be complicated by metabolic syndrome, cardiovascular diseases (e.g. myocardial infarction, stroke, hypertension) and other multisystem diseases. Available studies suggest that it is a result of excessive proliferation and abnormal differentiation of epidermal cells caused by excessive activation of immune cells (94). Its causes and may be related to genetic and environmental factors. Existing treatments are mainly aimed at relieving symptoms and have mixed results, and are not curative. A number of researchers have focused on the possibility of stem cell therapy for psoriasis (95). Meng et al. investigated the anti-inflammatory effects of DPSCs in a psoriasis-like mouse model established by imiquimod cream application. They found that intravenous infusion of DPSCs significantly decreased psoriasis-like pathological changes such inflammatory infiltration, epidermal thickening, hyperkeratosis, and hyperkeratosis in the skin of mice, and effectively improved the symptoms of psoriasis-like erythema, scaling, and thickening in the mice’s dorsal skin (96). The therapeutic effect of DPSCs on psoriasis was mainly achieved by reducing the inflammatory response. DPSCs treatment significantly downregulated T-bet (Th1 transcription factor) DPSCs treatment significantly downregulated the expression levels of T-bet (Th1-associated cytokine), IFN-γ (Th1-associated cytokine), RORγt (Th17 transcription factor), IL-17A, IL-17F and IL-23 (Th17-associated cytokine), and upregulated the expression levels of GATA3 (Th2 transcription factor), Foxp3 (Treg transcription factor) and IL-10. The mechanisms associated with DPSCs for the treatment of psoriasis lie in the down-regulation of Th1 and Th17 cell activity, as well as the promotion of Treg cell differentiation and the attenuation of the inflammatory response. HGF is mainly secreted by mesenchymal cells and helps to reduce the inflammatory response (97). The role of HGF was also verified by the work of Meng et al. Overexpression of HGF enhanced the inhibitory effect of DPSCs on Th1 and Th17 cell activity and the promotive effect of DPSCs on Treg cell activity (96).

## 7 Anti-inflammatory role of DPSCs in oral and other diseases

With their ease of access from teeth and better tissue repair/regeneration potential, DPSCs have gained a unique position in the field of regenerative dentistry (98). In the pulp tissue, the inflammatory and regenerative reactions are closely linked. Trauma or caries can trigger inflammation as well as regenerative effects at the molecular, cellular or tissue level in the dentin-pulp complex (99). As caries progresses and pulp structures become involved, the organism begins by fighting infection through an immune inflammatory response, followed by the recruitment of DPSCs that form the dentin-pulp complex for regeneration of lost portions of pulp structures including soft pulp tissue structures with vascular and neural components (100). Smaller and relatively slow injuries lead to dentin formation that compartmentalizes the injury from the pulp, whereas larger and rapidly progressive injuries lead to a more intense immune response in the pulp tissue, with clinical manifestations of pulpitis that can progress to pulp necrosis up to periapical periodontitis and alveolar bone inflammation (99).

Some current investigators have proposed that there are two main types of DPSCs to combat oral-associated inflammation, one relying on resident DPSCs in the pulp itself being stimulated, i.e., occurring during the process described above; the other can be transplanted into the root canal by ex vivo cultured DPSCs alone or in combination with appropriate scaffold material (101). When inflammation occurs, resident DPSCs are thought to exhibit opposing immunomodulatory and immunosuppressive effects at multiple cellular levels, particularly on DCs, macrophages and lymphocytes, reducing the amount of pro-inflammatory cytokines while locally increasing the amount of anti-inflammatory biomolecules (102). Meanwhile, inflammation-associated cytokines and growth factors significantly enhanced the proliferation, recruitment, and differentiation of DPSCs, which provided favorable conditions for further DPSCs to resist inflammation (103). Controlled activation of the intracellular p38MAPK and NF-KB signaling pathways in DPSCs during low-grade or short-term inflammation stimulates pulp repair or regenerative responses (104, 105). In the late stage of pulpal immunopathology, DPSCs control the inflammatory process together with endothelial cells, pulp fibroblasts, inflammatory cells from the peripheral circulation, and chemoattractive inflammatory cells (106). In conclusion, DPSCs directly or indirectly modulate the immune response to the anti-inflammatory phase (107).

Neves et al. found an increase in DPSCs when overlayed with Wnt agonist small molecules and an increase in macrophages near Wnt-receiving cells, indirectly regulating the anti-inflammatory state of macrophages from M1 to M2, with a possible molecular interaction between these two cell types (108). This suggests that Wnt/β-catenin signaling has a dual role in promoting restorative dentin formation by activating DPSCs and promoting anti-inflammatory macrophage responses, with a dual role in promoting restorative dentin formation (109). Fyn is a member of the protein tyrosine kinase family, whose overexpression is associated with various types of inflammation. Fyn forms a complex with Neuropilin-1, which inhibits adult dentin cell differentiation and amplifies inflammatory responses through the NF-κB signaling pathway. Fyn is involved in inflammatory and autoimmune disease imbalance, Fyn expression is reduced during adult dentin cell differentiation in DPSCs, where probably miR-125a-3p plays an important role in regulating its signaling pathway (110).

Periodontitis is a chronic inflammatory disease that leads to the destruction of alveolar bone and eventually to tooth loss (111). In genetically or environmentally susceptible individuals, periodontal pathogens trigger an inflammatory immune response in which activated macrophages secrete inflammatory cytokines and Th17 cells produce IL-17, NF-κB receptor activator ligand and TNF-α. The use of DPSCs offers the possibility to simultaneously target the inflammatory response to slow or stop its progression and to promote the regeneration of periodontal structures (63). Severe inflammation, progressive cartilage and bone destruction are typical pathological changes in temporomandibular joint arthritis, which makes treatment very difficult. Activation of immune cells and elevated expression of multiple inflammatory factors are closely associated with the development of inflammatory osteoarthritis (112, 113). Cui et al. used intra-articular drug injections to mimic the synovial inflammation and cartilage degradation of the temporomandibular joint. They then injected DPSCs locally and systemically, and discovered that local administration of the drug was superior to systemic administration in terms of reducing the progression of temporomandibular joint arthritis in rats by modifying the local immune-inflammatory response and cartilage matrix metabolism (114). Inhibition of the JAK-STAT1 pathway effectively downregulates immune cell activation and provides a target for DPSCs-based therapies (115). It was elaborated in their study that DPSCs effectively inhibit STAT1 pathway activation, leading to the downregulation of MMP3 and MMP13 (114).

## 8 Therapeutic effects of exosomes from DPSCs on inflammation associated with various diseases

The therapeutic potential of adult stem cells is highly dependent on the release of molecules and factors in the extracellular environment (116). DPSCs secrete soluble factors (proteins, lipids and nucleic acids) and extracellular vesicles through paracrine activity (117). Exosomes (EXOs) are a type of extracellular vesicles and are the main form of paracellular secretion. EXOs are small, double-layered, membranous lipid vesicles containing a variety of contents such as proteins and nucleic acids, with diameters ranging from 30 to 150 nm, involved in intercellular communication, promoting tissue repair and regeneration (118, 119). The morphology and structure of the EXOs that DPSCs secrete are not substantially different from those of other cell sources, and they maintain the same biological properties and functions as DPSCs. However, DPSCs-EXOs have the advantage of being more stable and easier to preserve than DPSCs (120).

Current studies suggest that the therapeutic effects of DPSCs are mainly attributed to their release of paracrine factors (121). As one of the most important paracrine mediators, DPSCs-derived EXOs show therapeutic effects through immunomodulation (122). Pivoraitė et al. injected SHED-EXOs intravenously into a mouse model of carrageenan-induced inflammation and found that SHED inhibited the activity of tissue proteinase B and matrix metalloproteinases at the site of acute inflammation, demonstrating for the first time that DPSCs can inhibit the carrageenan-induced acute inflammatory response in mice (123). In studying the protective effects of DPSC- EXOs against cerebral ischemia-reperfusion injury, Li et al. found that DPSC- EXOs inhibited neuro-inflammatory responses in brain cerebral ischemia-reperfusion mice by reducing the protein expression of IL-6, IL-1β and TNF-α through the HMGB1/TLR4/MyD88/NF-κB pathway (124). In another study, researchers established a periodontitis mouse model followed by local injection of DPSC- EXOs. They demonstrated that in mice with periodontitis, DPSC-EXOs conjugated chitosan hydrogel promoted the transformation of periodontal macrophages from a pro-inflammatory to an anti-inflammatory phenotype. The treatment also reduced periodontal damage by immune response modulation and suppression of periodontal inflammation, which sped up the healing of alveolar bone and periodontal epithelium in periodontitis mice through a mechanism that may be connected to miR-1246 in DPSC-EXOs (125).

The application of DPSC-EXOs does not require the direct use to cells, thus avoiding the limitations and risks associated with cell transplantation. Although DPSC-EXOs may face some limitations (heterogeneity, shortcomings of purification methods and tentative insurmountable large-scale production capacity), they are not only promising drugs, but may become effective vectors by engineering treatments that will provide particular ideas for the treatment of some diseases (15).

## 9 Therapeutic effects of conditioned media of DPSCs on inflammation associated with various diseases

As mentioned above, the paracrine role of DPSCs can express their therapeutic capacity to some extent. DPSCs secrete a large number of nutritional and immunomodulatory factors (126). Conditioned medium of DPSCs (DPSCs-CM) contain growth factors, cytokines and other active substances that mimic the regulatory effects of DPSCs on immunologically active cells. Conditioned media from various stem cell types have been shown to have considerable potential in the treatment of a variety of refractory diseases (127).

Temporomandibular joint osteoarthritis is a degenerative joint disease characterized by progressive cartilage degeneration, abnormal bone remodeling, and chronic pain (128). When Ogasawara et al. administered SHED-CM intravenously to mice in a model of temporomandibular joint osteoarthritis brought on by mechanical stress, they saw a decline in the number of chondrocytes expressing IL-1β, iNOS, and MMP-13, a significant reduction in temporal muscle inflammation, and a contribution to the regeneration and repair of the mice’s osteoarthritis (129). Rheumatoid arthritis is an autoimmune disease characterized by synovial hyperplasia and chronic inflammation leading to progressive destruction of articular cartilage and bone (130). The work of Ishikawa et al. by a single intravenous injection of SHED-CM into a mouse model of anti-collagen-type antibody-induced arthritis in rheumatoid arthritis-like mice yielded results that SHED-CM has therapeutic anti-inflammatory effects, ameliorating arthritic symptoms and inhibiting tissue damage by shifting the pro-inflammatory synovial environment to an anti-inflammatory synovial environment through induction of M2 macrophage polarization (131). As mentioned earlier, Alzheimer’s disease is a progressive neurodegenerative disease characterized by cognitive decline and the presence of β-amyloid plaques in the brain. The disease occurs when activated microglia release various neurotoxic factors, including pro-inflammatory cytokines (132). In a study conducted on a mouse model of Alzheimer’s disease, Mita et al. used SHED-CM and discovered that it reduced the pro-inflammatory response brought on by β-amyloid plaques and changed the pro-inflammatory M1-type microglia microenvironment into an M2-type anti-inflammatory/neuro-protective microenvironment (133). As was previously mentioned, anti-inflammation may be a crucial component of PNI treatment. The study by Fumiya Kano et al. demonstrated that SHED-CM is a crucial component of PNI treatment through monocyte chemoattractant protein-1 and the secreted ectodomain of sialic acid-binding Ig-like lectin-9. This allowed for the induction of tissue-repairing M2 macrophages and the promotion of facial nerve function recovery in rats following injury, confirming that the anti-inflammatory effect of SHEDs can have a therapeutic effect on severe PNI (134). Similarly, severe inflammation impedes functional recovery after SCI. Matsubara et al. injected SHED-CM intrathecally into the injured spinal cord of a rat model during the acute period of SCI and found that SHED-CM induced immunomodulatory activity of anti-inflammatory M2-like macrophages, thereby promoting axonal growth, peripheral neural tissue angiogenesis, schwann migration, proliferation and activation, and neuronal survival, leading to significant functional recovery, similar to the effect of direct SHEDs transplantation (135). Acute liver failure occurs with massive hepatocellular destruction and an intense inflammatory response (136). In a study by Matsushita et al., SHEDS or SHED-CM were intravenously injected into ALF rat models. The results showed attenuation of the pro-inflammatory response and induction of anti-inflammatory M2-like hepatic macrophages, proving that the anti-inflammatory effect of SHED helps create a favorable environment for tissue regeneration after ALF (137). Hepatocyte necrosis and apoptosis, which arise from chronic liver injury, then activate pro-inflammatory mediators and encourage the transdifferentiation of hepatic stellate cells into myofibroblasts, which leads to the development of liver fibrosis. Severe irreversible liver fibrosis is followed by liver failure (138). The work of Hirata et al. in which SHED-CM was injected into mouse model of liver fibrosis, inhibited gene expression of pro-inflammatory mediators, protected parenchymal hepatocytes from apoptosis, induced apoptosis in activated hepatic stellate cells, and induced apoptosis by inducing expression of MMP13 polarization of macrophages to promote fibrinolysis, ultimately leading to the regression of fibrous scarring (139). Ischemic heart diseases such as myocardial infarction can cause irreversible damage to the heart. Yamaguchi et al. showed that SHED-CM significantly reduced endotoxin-induced expression of pro-inflammatory genes, decreased the area of myocardial infarction after ischemia/reperfusion, decreased cardiomyocyte apoptosis under ischemic and hypoxic conditions, and decreased the levels of inflammatory cytokines in a mouse ischemia/reperfusion (I/R) model (140). Sjogren’s syndrome (SS) is a chronic systemic autoimmune disease with a complex pathogenesis involving multiple inflammatory cells and inflammatory factors. Compared to other inflammatory autoimmune diseases, including Rheumatoid arthritis, blocking TNF-α has little effect on SS patients (141). Kawashima et al. used DPSC-CM in a mouse SS model to promote the differentiation of Treg cells and inhibit the differentiation of Th17 cells in the spleen of mice, which reduced the inflammation of submandibular gland and improved the symptoms of SS (142). Ogata et al. also used DPSC-CM in a mouse SS model and obtained the same conclusion (143). As mentioned earlier, ARDS is a severe inflammatory disease characterized by acute respiratory failure caused by severe destructive pulmonary inflammation and irreversible pulmonary fibrosis (74). Wakayama et al. Subjected to ALI model mice, a single intravenous injection of SHED-CM attenuated the pro-inflammatory response and produced an anti-inflammatory/tissue regenerative environment, while inducing anti-inflammatory M2-like lung macrophages, which facilitated the treatment of lung injury (144).

The application of DPSC-CM/SHED-CM does not require direct use to the cells, similar to EXOs, avoiding risks such as potential tumorigenicity associated with direct use of the cells themselves, while not requiring the tedious and labor-intensive processing required to extract EXOs. This provides another exceptional idea for the treatment of multiple refractory diseases.

## 10 Mechanisms related to the anti-inflammatory and immunomodulatory effects of DPSCs

Like other MSCs, DPSCs can elude immune identification and inhibit immunological responses and are considered less immunogenic, probably because of the low expression levels of major histocompatibility complex class I molecules and the negativity of major histocompatibility complex class II cells (145, 146). Perhaps there are opportunities to use DPSCs’ immunosuppressive characteristics as a therapeutic immunomodulatory tool for the management of inflammatory and other inflammation-related autoimmune diseases, and even more so for the adjuvant management of allograft rejection and graft-versus-host disease (147).

**Figure 3:**
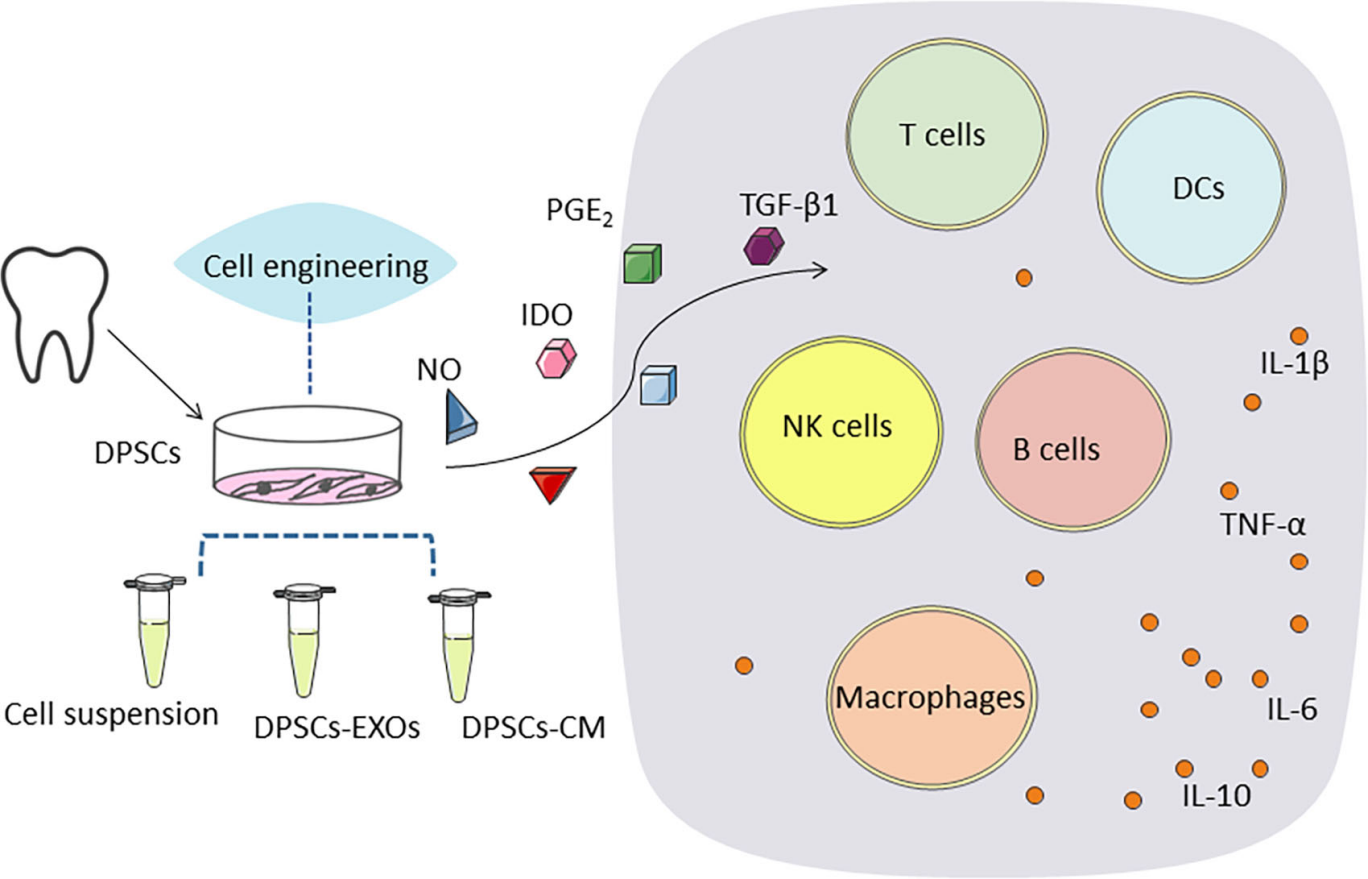
DPSCs secrete soluble factors mainly including, nitric oxide (NO), indoleamine 2,3-dioxygenase (IDO), prostaglandin E2 (PGE2) and transforming growth factor β1 (TGF-β1), which help to mediate immunosuppression. DPSCs stimulate regulatory T cell (Treg) function and inhibit all other cell types of the immune system, such as B and T cells, monocytes, dendritic cells. Gene transfer of an immunomodulatory or other factor may further enhance the efficacy of DPSCs therapy. DPSCs promote regeneration and healing by up-regulating anti-inflammatory immune cells and associated cytokines and down-regulating pro-inflammatory immune cells and associated cytokines to inhibit excessive inflammatory responses.

As previously mentioned, several existing studies have addressed the anti-inflammatory effects and immunomodulatory capacity of DPSCs. DPSCs have a strong immunomodulatory capacity, either through intercellular contacts or through some of their secreted components, which can inhibit proliferation, reduce cytokine and antibody secretion, control immune cell maturation, and interfere with antigen presentation by T cells, B cells, NK cells, and DCs (148, 149). The main mechanism associated with the therapeutic effects of DPSCs is the indirect effect of the release of various cytokines, growth factors and chemokines through paracrine action (150). Available studies have pointed out that they secrete soluble factors mainly including HGF, nitric oxide, indoleamine 2,3-dioxygenase (IDO), prostaglandin E2 and TGF-β1, which help to mediate immunosuppression (151–153) (**Figure 3**).

Lymphocytes are an important component of the immune system and consist mainly of T cells, B cells, NK cells and DCs. Among them, T cells and B cells are capable of producing specific immune responses to pathogens and become a major component of the adaptive immune system in addition to innate immunity (154). T-cell responses occur when antigen-presenting cells induce and control the differentiation of T cells. Initial CD4+ T helper cells may differentiate and transform into helper T cells1 (Th1), Th2, Th17 and Treg cells (155, 156). IL-17 induces fibroblasts, endothelial cells, macrophages and epithelial cells to produce pro-inflammatory cytokines, such as IL-1, IL-6 and TNF-α and certain chemokines, which generate and maintain an inflammatory environment (157, 158). Treg induces the secretion of IL-10 and TGF-β, which are used to regulate the immune response and suppress inflammation (159). B cells produce antibodies and interact closely with T cells, resulting in a variety of autoimmune diseases. Among soluble factors, TGF-β, HGF, prostaglandin E2 and IDO are thought to play an important role in MSCs-mediated B-cell immunosuppressive activity (160). The two most significant specialized antigen-presenting cells, DCs and macrophages, are essential for T-cells activation and the polarization of the adaptive immune response. Typically, macrophages are categorized as pro-inflammatory M1-type cells or anti-inflammatory M2-type cells (161, 162). M1 cells initiate inflammation by releasing high levels of pro-inflammatory cytokines, glutamate, reactive oxygen species, and nitric oxide. Correspondingly, M2 cells counteract inflammation and promote tissue repair and regeneration by secreting anti-inflammatory cytokines, phagocytic debris, and promoting tissue repair and regeneration (163). According to a study by Albashari et al., DPSCs significantly modulated the expression of NF-κB and its inhibitor IκB-α, attenuated the effect of lipopolysaccharide-induced polarization of inflammatory macrophages RAW 264.7 toward the M2 type, and decreased the expression of IL-6, which is achieved through the NF-κB signaling pathway (33). This has been the most likely effect of DPSCs in inflammatory diseases that many researchers have focused on, i.e., controlling the polarization of macrophages, inhibiting pro-inflammation and enhancing anti-inflammation.

Like Bone marrow mesenchymal stem cells, DPSC may inhibit T cell proliferation by secreting IDO induced by IFN-g (164). Recruitment of activated t cells by secreting fas (a death receptor known as tumor necrosis factor receptor superfamily member 6)-mediated monocyte chemoattractant protein-1 ensures intercellular contact, which in turn induces t cell apoptosis. Apoptotic t cells subsequently trigger the production of high levels of TGF-β by macrophages, which in turn leads to upregulation of CD4+CD25+Foxp3+ Tregs, thereby inducing the final immune effect (70). It has been shown that DPSCs can induce apoptosis of already activated T cells *in vitro* (69). DPSCs can inhibit the proliferation of stimulated T cells, and this inhibition is actually stronger than that of bone marrow mesenchymal stem cells (165). NK cells are important effector cells of innate immunity and play a key role in antitumor and antiviral effects through their cytotoxic and proinflammatory cytokines, including TNF-α and IFN-γ secretion. NK cells are also closely associated with inflammatory diseases, such as inflammatory bowel disease and periodontitis, among others (166). Regulation of B cells and NK cells by MSCs blocks pathogenic inflammatory and immune responses *in vivo* (167). The potential mechanism of DPSCs anti-inflammation is also closely related to the inhibition of NK cell proliferation, reduction of cytotoxicity, and promotion of apoptosis (168). DCs are the most potent antigen-presenting cells and play a key role in the effects of immunity and tolerance depending on the activation and maturation phases, as well as on the cytokine milieu at the site of inflammation (169). Mechanisms of DPSC anti-inflammation and immunomodulation may be related to interfering with the differentiation, maturation and function of DCs (170, 171). FasL is a transmembrane protein whose expression plays an important role in the induction of the Fas/FasL apoptotic pathway. In *in vitro* experiments, knockdown of FasL expression in DPSCs by siRNA led to a reduction in T cell apoptosis, making the anti-inflammatory effect of DPSCs greatly reduced which implies that FasL regulates the immunomodulatory properties of DPSCs in the context of inducing T cells apoptosis (69). DPSCs may be an effective treatment not only in oral inflammation, but in a variety of other acute and chronic inflammatory conditions.

As mentioned above, because DPSCs originate from the dental pulp, the pulp itself may be attacked by inflammation, complicating the relationship between inflammation and DPSCs. Inducing cell proliferation in DPSCs through chronic inflammation mediated by inflammatory biomolecules like TNF-α and C3a has been demonstrated; these proliferation-inducing effects are linked to NF-κB intracellular signaling stimulation, which may be related to the activation of the typical Wnt/β-Catenin pathway, while the atypical Wnt/Ca2+ pathway is inhibited (172, 173). When this stimulatory inflammatory challenge exceeds the capacity of the pulp tissue, the positive effect is diminished or even overridden. Stimulation by IFN-γ significantly upregulates the migratory capacity of DPSCs (173). Enhanced research in this area may provide more possibilities for DPSCs treatment of inflammatory diseases.

During inflammation caused by some bacterial injuries, NF-κB and p38MAPK intracellular signaling pathways are activated, releasing a large number of inflammation-associated biomolecules including IL-1α, IL -1β, IL-4, IL-6, IL-8, IL-10, TNF-α and a series of inflammatory molecular mediators (174). Neutrophils, macrophages, antigen-presenting DCs, T cells and later B cells are recruited and activated, producing a very complex effect. Multiple immune cells, in turn, produce additional inflammatory cytokines and antibodies to fight bacterial attack (151). Through their immunomodulatory effects, DPSCs play a decisive role in anti-inflammatory control through cell-cell contact and secreted substance mediation, especially IDO and TGF-β1 (33, 175). Through their action, DPSCs inhibit proliferation, maturation, antigen presentation and antibody/cytokine production of inflammatory cells (176).

## 11 Summary

In addition to their well-established self-renewal and pluripotent differentiation properties, MSCs also possess powerful anti-inflammatory and immunomodulatory functions *in vitro* and *in vivo*, making them candidates for the treatment of a variety of inflammation-related and autoimmune diseases (176). As a member of MSCs, DPSCs are favored by many researchers because of their easier availability and superior immunomodulatory ability than other MSCs (22, 176). The potentiality of DPSCs in the treatment of neurological, pulmonary, hepatic, renal, intestinal, skin-related inflammatory and autoimmune diseases should not be underestimated, probably because they themselves or some substances they secrete inhibit T cells proliferation and function through inflammatory immune-related signaling pathways, stimulate Treg cells, inhibit DCs differentiation, and convert macrophages to an anti-inflammatory phenotype role (103, 177) (**Table 1**).

**Table 1:** Examples of the anti-inflammatory effects of DPSCs in animal models.

Cell Type	Disease Model	Organs/Tissues	Administration Mode	Outcome	Effect Evaluation And Safety Assessment	References
human DPSCs	Peripheral nerve injury rat model, SD rats	nerve	1×10^6^/20µL DPSCs were transplanted	Iba1, ROS, NF-κB ↓ pAMPK/SIRT1↑ IL-1β,TNF-α ↓ IL-4,TGF-β↑	DPSCs attenuate inflammation of the supraspinal nerve caused by oxidative stress and homeostatic dysregulation after sciatic nerve injury.	(12)
human DPSCs	Acute spinal cord injury model, SD rats	nerve	DPSCs (1×10^6^cells/mL) were mixed in hydrogels, *in situ* injection of 10 μL	IL-6,TNF-α ↓ NF-κB,IκB-α↓	DPSCs were effective in controlling inflammation in SCI rats, thus greatly promoting neural repair.	(33)
human DPSCs	3-nitropropionic acid rat model of Huntington’s disease, SD rats	brain	After 2 days, 2.5 × 10^5^ DPSCs was suspended in 2μL culture media, transplanted in the Medio-posterior part of the striatum	TNF,IL-1β, ↓	DPSCs reduce the expression of inflammatory cytokines in the striatum and facilitate the treatment of Huntington’s chorea.	(48)
DPSCs of SD rats	STZ-induced diabetic polyneuropathy, SD rats	sciatic nerve	After 8 weeks, Skeletal muscle injection (1mL in total, 1×10^6^ cells)	TNF-α ↓ IL-10,CD206↑	DPSCs could be an efficacious anti-inflammatory cell therapy for diabetic polyneuropathy by modulating the proportions of M1/M2 macrophages.	(55)
human DPSCs	Hypoxia-Ischemia rat model, SD rats	brain	1 × 10^6^ DPSCs in 10 μL PBS, stereotactic injection into the subdural cortical area	IL-1β,IL-6,TNF-α,INF-γ↓ IL-10, TGF-β↑	Implantation of autologous DPSCs reduced the inflammatory response in the NHI brain and promoted neuronal growth, differentiation and regeneration.	(57)
DPSCs of SD rats	Dextran Sulfate Sodium -induced mouse colitis, C57BL/6 mice	colon	After 3 days, 1 x 10^5^ cells/10g body weight in 100 μL PBS, infused into mice		DPSCs were capable of ameliorating inflammatoryrelated tissue injuries when systemically infused into a murine colitis model.	(69)
human DPSCs	Paraquat Model, C57BL/6J mice	lung	After 1 or 3 days,1 × 10^6^DPSCs in 200 μL of saline, injected into the tail vein	IL-1β,IL-6,TNF-α↓	HGF-modified DPSCs have a robust ability to modulate inflammation by suppressing lung inflammation to treat paraquat -induced lung injury.	(77)
SHEDs	Ovalbumin induced model of allergic airway inflammation, BALB/c mice	lung	4 × 10^6^ DPSCs via tail vein injection		Mice treated with DPSCs displayed a significant inhibition of allergic airway inflammation.	(79)
human DPSCs	COPD model, C57BL/6 mice	lung	After two weeks, intratracheal injection of 5*10^5^ fifth generation DPSCs in 50 µL PBS	IL-1β,IL-6,TNF-α↓	DPCSs can reduce inflammation and oxidative stress in COPD. DPSCs may inhibit inflammation and oxidative stress by increasing the expression of Nrf2 and its downstream factors.	(83)
DPSCs	lupus mice model, B6.MRL-Faslpr/J mice	kidney	DPSCs were resuspended in PBS and intravenously infused at 2×10^5^ per 10 g body weight into mice.	IL-6,IL-10,IL-17↓	Transplantation of DPSCs ameliorates nephritis in lupus mice.	(91)
human DPSCs	imiquimod cream BALB/C mice	skin	200μL normal saline suspension of 2×10^6^ DPSCs, intravenous tail injection	IFN-γ,TNF-α,IL-17A↓	HGF overexpression enhanced the treatment effect of DPSCs on psoriasis by reducing inflammatory responses.	(96)

DPSCs, dental pulp stem cells; SHEDs, stem cells from human exfoliated deciduous teeth; SD rats, Sprague−Dawley rats; SCI, Spinal cord injury; HGF, hepatocyte growth factor; NHI, Neonatal hypoxia-ischemia; IL, interleukin; TNF, tumor necrosis factor; IFN, interferon; TGF, transforming growth factor; ROS, reactive oxygen species; NF-κB, Nuclear Factor-κ-gene Binding; Iba, indole butyric acid; PBS, phosphate buffered saline.

**Figures 1**, **2** were created with MedPeer (www.medpeer.cn).

"↑", means increase; "↓", means decline.

Many countries have set up institutions specializing in the collection of DPSCs, and they have well-established processes for accessing DPSCs, including collection, transportation, stem cell isolation and culture, and cryogenic storage (178). Stockpiled DPSCs can be used in the future for both their personal use as well as for family members as necessary because of the low immunogenicity of DPSCs. Nagpal et al. administered appropriate concentrations of autologous DPSCs from elderly people into the infarct foci and surrounding areas of stroke survivors with moderate to severe disabilities using intracranial injections (179). After conducting a number of preoperative and postoperative observations and analyses, they concluded that autologous DPSC transplantation for the treatment of chronic symptoms following stroke was safe and practicable (179). Allogeneic DPSCs were used in a clinical trial by Ye et al. to treat severe COVID-19. They confirmed the safety and effectiveness of allogeneic DPSC transplantation by contrasting experimental and control groups in a limited sample size (180). Li et al. infused SHED in type 2 diabetes patients receiving insulin, and the outcomes demonstrated that SHED infusion is a secure and reliable treatment that enhances islet function and glucose metabolism in type 2 diabetes patients (181). The therapeutic potential and storage value of DPSCs are anticipated to get an increasing amount of attention as their safety and efficacy have been widely established.

However, as mentioned in this paper, DPSCs are derived from pulp tissue, which itself may experience inflammatory stimuli while in the oral cavity or during the process of being acquired, and furthermore, the means of culturing DPSCs, the environment, and the manner and method of delivering DPSCs or its products to the organism may affect the functional effects of DPSCs (3, 17). IFN-γ, TNF-α and IL-1β infusion into DPSCs may enhance their immunosuppressive capacity and can be considered as a feedback mechanism to suppress the exacerbated immune response (20). The role of the interface between DPSCs and immune cells is complex, and the roles of leader and leaderee may be interchanged under specific conditions (151). To really explain it in depth this is still a problem that needs to be overcome. Perhaps we can use such relationships to purposefully engineer DPSCs in order to obtain the drugs we are targeting to treat various diseases. For example, stimulating DPSCs to release engineered extracellular vesicles loaded with specific molecular cargoes for targeted delivery to cells and tissues treated with the ligand of interest (182).

We expect more research to be conducted on DPSCs, not only to achieve results in the laboratory and animal models, but also to serve clinical needs and transform them into practical tools that can truly contribute to the treatment of diseases and human health.

## Author contributions

FL: Writing – original draft, Writing – review & editing. XW: Writing – review & editing. JS: Writing – review & editing. SW: Writing – review & editing. WX: Writing – review & editing. YH: Writing – review & editing.
